# Data on metals biomonitoring in the body of schoolchildren in the vicinity of a heavily industrialized site

**DOI:** 10.1016/j.dib.2017.04.027

**Published:** 2017-04-27

**Authors:** Raheleh Kafaei, Rahim Tahmasebi, Masomeh Ravanipour, Dariush Ranjbar Vakilabadi, Mehdi Ahmadi, Mohammad Reza Farzaneh, Bahman Ramavandi

**Affiliations:** a Department of Environmental Health Engineering, Faculty of Health and Nutrition, Bushehr University of Medical Sciences, Bushehr, Iran; b Department of Biostatistics, Faculty of Health and Nutrition, Bushehr University of Medical Sciences, Bushehr, Iran; c Environmental Technologies Research Center, Ahvaz Jundishapur University of Medical Sciences, Ahvaz, Iran; d Department of Environmental Health Engineering, Ahvaz Jundishapur University of Medical Sciences, Ahvaz, Iran; e Department of Pathology, Faculty of Medicine, Bushehr University of Medical Sciences, Bushehr, Iran

**Keywords:** Biomonitoring, Metals, Schoolchildren, Urine, Asalouyeh city

## Abstract

This data is obtained from analyzing the concentration of metals include Al, Co, Cr, Cu, Fe, Mo, Pb, and Zn in the urine of schoolchildren in Asalouyeh city in vicinity to a heavily industrialized site and comparison with a reference city. The significance of sex groups on urine metal level was evaluated through this data. The urinary content of metals was measured by inductively coupled plasma atomic emission spectroscopy (ICP-OES). Statistical analyze of data were done by Mann–Whitney test. The herein presented date could beneficial for health assessment of gas and petrochemical companies.

**Specifications Table**

**Table:** 

Subject area	*Environmental science*
More specific subject area	*Environmental epidemiology, toxicology*
Type of data	*Table*
How data was acquired	*ICP-OES (SPECTRO, Spectro arcos, Germany)*
Data format	*Analyzed*
Experimental factors	*Urine samples were collected from schoolchild and were frozen until analysis. After defrosting and pretreatment, the metals level of the samples was analyzed.*
Experimental features	*Measurement of 8 metals (*Al, Co, Cr, Cu, Fe, Mo, Pb, and Zn) *concentration in schoolchildren*.
Data source location	*Asalouyeh and Saadabad city in Bushehr province, Iran.*
Data accessibility	*Data is presented with the article*

**Value of the data**

• Data is useful to assess the gas and petrochemical sites effects on sensitive populations such as schoolchildren close them.
• Data reflect the human biomonitoring results as a tool to assess human exposure to environmental pollution.
• Data show the urine metal properties could serve as a bioindicator in human biomonitoring.

## 1 Data

The concentration levels of urinary metal summarized in Table 1, Table 2. Table 1 shows the concentration of metal measured in two areas. Detection limits (LOD) of each metal is also shown in Table 1. Table 2 presents data in sex groups in polluted area. All metal levels are presented as ppb unit.

## 2 Experimental design, materials and methods

### 2.1 Study groups and field study

The field study was conducted in April 2015. The participants were 6–12 year old children in Asalouyeh city (as polluted area) and Saadabad city (as reference area). Both areas are located in Bushehr province, Iran with the same population and same elementary schoolchildren population that attending to a boys school or a girls school. In each area 20 cases randomly selected between who had been living in the area for at least 3 consecutive years. Finally 40 samples were collected for this study (20 boys and 20 girls). A questionnaire was prepared containing socio-demographic characteristics, health status and medication and tobacco smoking (active and passive). The proposal was approved by Bushehr University of medical science.

### 2.2 Urine sample and chemical analysis

A spot urine sample was collected using a 100 ml sterile polystyrene container. The samples were frozen at −20 °C then, before analyzing samples were defrosted at 4 °C, homogenized, filtered and placed in polyethylene tubes pre-treated with dilute nitric acid and rinsed with distilled water. The metals of Al, Co, Cr, Cu, Fe, Mo, Pb, and Zn were quantified in all samples by using ICP-OES [1], [2].

### 2.3 Statistical analysis

Statistical analyses were conducted using the Statistical Package for the Social Sciences software (SPSS version 22). The arithmetic mean (AM), Geometric mean (GM), minimum value, maximum value, range and percentiles were calculated. Mann–Whitney test was used to evaluation of significance differences between variables.

## Figures and Tables

**Table 1:** Urinary levels of metals in Asalouyeh and reference schoolchildren (ppb).

Metal	Place	*N*	LOD	AM (SD)	GM	Min	P25	Med	P75	P95	Max	Range	*P*_value_
Al	Polluted	20	1.00	134.84 (470.72)	<0.001	<0.001	3.32	7.90	23.62	2022.31	2113.20	2113.20	0.098
Al	Reference	20	1.00	103.11 (362.93)	<0.001	<0.001	0.20	2.80	13.90	1543.85	1605.70	1605.70	
Co	Polluted	20	0.1	2.36 (2.00)	1.95	0.90	1.22	1.70	2.57	9.72	10.00	9.10	0.249
Co	Reference	20	0.1	1.76 (.91)	1.57	0.80	1.02	1.50	2.27	3.97	4.00	3.20	
Cr	Polluted	20	1.00	2.44 (3.49)	<0.001	<0.001	0.05	1.15	2.67	11.26	11.30	11.30	0.014⁎
Cr	Reference	20	1.00	6.29 (5.35)	<0.001	<0.001	1.67	5.15	10.27	16.99	17.10	17.10	
Cu	Polluted	20	1.00	12.92 (7.23)	11.00	1.80	7.50	11.90	16.97	33.11	33.70	31.90	0.409
Cu	Reference	20	1.00	11.11 (5.44)	9.84	4.10	5.75	10.45	16.97	21.45	21.60	17.50	
Fe	Polluted	20	1.00	14.47 (10.51)	11.56	3.20	7.65	11.60	19.82	43.03	43.50	40.30	0.925
Fe	Reference	20	1.00	18.63 (23.23)	12.14	3.20	7.37	10.80	18.80	98.13	100.10	96.90	
Mo	Polluted	19	2.00	55.46 (38.13)	41.52	11.00	17.40	47.80	93.00	–	122.00	111.00	0.448
Mo	Reference	20	2.00	54.16 (37.62)	33.93	12.00	18.03	31.15	73.15	140.37	141.00	130.00	
Pb	Polluted	20	1.00	12.33 (19.53)	8.18	2.80	5.30	7.05	9.97	89.63	93.20	90.40	0.051
Pb	Reference	20	1.00	14.90 (10.11)	11.96	3.90	6.87	9.70	25.67	33.35	33.40	29.50	
Zn	Polluted	20	1.00	570.33 (491.92)	453.09	180.30	265.65	421.35	699.25	2295.12	2359.30	2179.00	0.058
Zn	Reference	20	1.00	356.02 (232.85)	296.08	108.60	172.12	334.70	446.00	1012.87	1024.00	915.40	

AM (SD): Arithmetic Mean (Standard Deviation), GM: Geometric mean, Px: Percentile x.

⁎ *p*<0.05.

**Table 2:** Urinary levels of metals in Asalouyeh children between sex groups (ppb).

Metal	Sex	*N*	AM (SD)	GM	Min	P25	Med	P75	Max	Range	*P*_value_
Al	Girl	10	19.85 (41.34)	<0.001	<0.001	0.82	5.95	14.85	134.00	134.00	0.059
Al	Boy	10	249.83 (660.82)	22.29	3.10	6.55	12.30	93.82	2113.20	2110.10	
Co	Girl	10	1.92 (0.78)	1.77	0.90	1.17	1.70	2.60	3.10	2.20	0.791
Co	Boy	10	2.80 (2.72)	2.15	1.10	1.27	1.80	3.07	10.00	8.90	
Cr	Girl	10	3.64 (4.59)	<0.001	<0.001	0.15	1.65	9.00	11.30	11.30	0.446
Cr	Boy	10	1.24 (1.21)	<0.001	<0.001	<0.001	1.05	2.62	3.00	3.00	
Cu	Girl	10	10.91 (4.75)	9.46	1.80	7.17	11.70	14.65	17.70	15.90	0.364
Cu	Boy	10	14.93 (8.87)	12.79	5.80	7.55	13.30	21.60	33.70	27.90	
Fe	Girl	10	8.82 (4.76)	7.71	3.20	4.80	7.80	12.50	18.10	14.90	<0.0018⁎
Fe	Boy	10	20.13 (11.82)	17.34	8.70	9.52	17.10	29.62	43.50	34.80	
Mo	Girl	10	37.58 (36.58)	26.16	11.00	12.75	18.05	55.13	122.00	111.00	0.027
Mo	Boy	9	75.32 (30.39)	69.36	35.00	46.55	69.60	104.90	115.00	80.00	
Pb	Girl	10	17.34 (27.15)	9.82	2.80	4.82	8.85	13.10	93.20	90.40	0.307
Pb	Boy	10	7.32 (3.46)	6.81	4.60	5.30	6.15	8.17	16.30	11.70	
Zn	Girl	10	577.47 (655.63)	412.66	180.30	262.85	293.35	669.42	2359.30	2179.00	0.326
Zn	Boy	10	563.19 (284.43)	497.46	191.70	342.10	512.85	772.37	1075.70	884.00	

AM (SD): Arithmetic Mean (Standard Deviation), GM: Geometric mean, Px: Percentile x.

* *p*<0.05.       **p<0.05.
